# Polygenic risk of major depressive disorder as a risk factor for venous thromboembolism

**DOI:** 10.1182/bloodadvances.2023010562

**Published:** 2023-07-06

**Authors:** Joey Ward, Ngoc-Quynh Le, Suryakant Suryakant, Jennifer A. Brody, Philippe Amouyel, Anne Boland, Rosemary Bown, Breda Cullen, Stéphanie Debette, Jean-François Deleuze, Joseph Emmerich, Nicholas Graham, Marine Germain, Jana J. Anderson, Jill P. Pell, Donald M. Lyall, Laura M. Lyall, Daniel J. Smith, Kerri L. Wiggins, José Manuel Soria, Juan Carlos Souto, Pierre-Emmanuel Morange, Nicholas L. Smith, David-Alexandre Trégouët, Maria Sabater-Lleal, Rona J. Strawbridge

**Affiliations:** 1 School of Health and Wellbeing, University of Glasgow, Glasgow, United Kingdom; 2 Genomics of Complex Disease Unit, Institut d’Investigació Biomèdica Sant Pau, Barcelona, Spain; 3 University of Bordeaux, INSERM, Bordeaux Population Health Research Center, UMR 1219, Bordeaux, France; 4 Cardiovascular Health Research Unit, Department of Medicine, University of Washington, Seattle, WA; 5 University of Lille, INSERM, CHU Lille, Institut Pasteur de Lille, U1167-RID-AGE-Facteurs de Risque et Déterminants Moléculaires des Maladies Liées au Vieillissement, Lille, France; 6 Université Paris-Saclay, CEA, Centre National de Recherche en Génomique Humaine, Evry, France; 7 Laboratory of Excellence in Medical Genomics, GENMED, Evry, France; 8 School of Cardiovascular and Metabolic Health, University of Glasgow, Glasgow, United Kingdom; 9 Centre d’Etude du Polymorphisme Humain, Fondation Jean Dausset, Paris, France; 10 Department of Vascular Medicine, Paris Saint-Joseph Hospital Group, University of Paris, Paris, France; 11 UMR1153, INSERM CRESS, Paris, France; 12 Centre for Clinical Brain Sciences, University of Edinburgh, Edinburgh, United Kingdom; 13 Unitat d’Hemostàsia i Trombosi, Institut d’Investigació Biomèdica Sant Pau, Hospital de la Santa Creu i Sant Pau, Barcelona, Spain; 14 Aix-Marseille University, INSERM, INRAE, Centre de Recherche en CardioVasculaire et Nutrition, Laboratory of Haematology, CRB Assistance Publique – Hôpitaux de Marseille, HemoVasc, Marseille, France; 15 Department of Epidemiology, University of Washington, Seattle, WA; 16 Kaiser Permanente Washington Health Research Institute, Kaiser Permanente Washington, Seattle, WA; 17 Department of Veterans Affairs Office of Research and Development, Seattle Epidemiologic Research and Information Center, Seattle, WA; 18 Cardiovascular Medicine Unit, Department of Medicine Solna, Karolinska Institute, Stockholm, Sweden; 19 Health Data Research UK, Glasgow, United Kingdom

## Abstract

Major depressive disorder (MDD), bipolar disorder (BD), and schizophrenia (SCZ) are associated with an increased risk of cardiovascular diseases, including venous thromboembolism (VTE). The reasons for this are complex and include obesity, smoking, and use of hormones and psychotropic medications. Genetic studies have increasingly provided evidence of the shared genetic risk of psychiatric and cardiometabolic illnesses. This study aimed to determine whether a genetic predisposition to MDD, BD, or SCZ is associated with an increased risk of VTE. Genetic correlations using the largest genome-wide genetic meta-analyses summary statistics for MDD, BD, and SCZ (Psychiatric Genetics Consortium) and a recent genome-wide genetic meta-analysis of VTE (INVENT Consortium) demonstrated a positive association between VTE and MDD but not BD or SCZ. The same summary statistics were used to construct polygenic risk scores for MDD, BD, and SCZ in UK Biobank participants of self-reported White British ancestry. These were assessed for impact on self-reported VTE risk (10 786 cases, 285 124 controls), using logistic regression, in sex-specific and sex-combined analyses. We identified significant positive associations between polygenic risk for MDD and the risk of VTE in men, women, and sex-combined analyses, independent of the known risk factors. Secondary analyses demonstrated that this association was not driven by those with lifetime experience of mental illness. Meta-analyses of individual data from 6 additional independent cohorts replicated the sex-combined association. This report provides evidence for shared biological mechanisms leading to MDD and VTE and suggests that, in the absence of genetic data, a family history of MDD might be considered when assessing the risk of VTE.

## Key Points

• Genetic regulation of MDD and VTE was correlated.
• Genetic predisposition to MDD was associated with an increased risk of VTE.

## Visual Abstract

**Figure:**
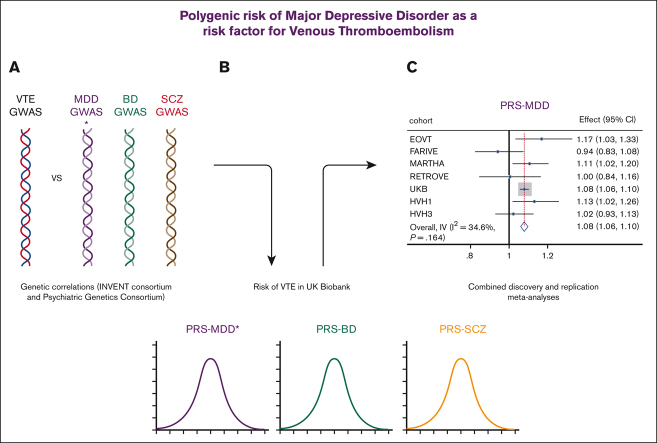

## Introduction

Individuals with severe mental illness (SMI, schizophrenia [SCZ], bipolar disorder [BD], and major depressive disorder [MDD]) have an increased risk for several metabolic and cardiovascular diseases,^1^ including venous thromboembolism (VTE).^2^^,^^3^

A systematic review demonstrated the increased risk of VTE in psychotic disorders, depression, and bipolar disorder^4^, with a shift in the balance of pro- and anticoagulation signals resulting in hypercoagulability being proposed as a mechanism.^2^ Smoking, obesity, pregnancy, and exogenous hormones have procoagulation effects, with the ABO blood group also influencing coagulation potential. Individuals with SMI have an increased burden of VTE risk factors, such as obesity, smoking,^2^ sedentary behavior^5^ with antipsychotic and antidepressant medication, and immobility (for example, during catatonia or sedation)^2^ also increasing the risk. Further evidence of increased thrombotic risk includes altered levels of procoagulant factors in patients with SMI: altered serotonin, which is a key component of MDD, and an activator platelet;^3^ elevated D-dimer, which is a marker of prothrombotic events; elevated coagulation factor VIII in patients with first-episode psychosis;^3^ and reduced anticoagulant protein S levels observed in SCZ.^3^ Platelets are particularly interesting, as they demonstrate functional similarities with neurons,^6^ and their levels or activity have been shown to be altered in MDD^6^ and by serotonin–affecting antidepressant medication.^7^

The Psychiatric Genetics Consortium has collated sufficiently large sample sizes and genome-wide association studies (GWAS) have identified risk variants for SCZ,^8^ BD,^9^ and MDD.^10^ In parallel, GWAS of endophenotypes/characteristic features of SMI (such as anhedonia^11^ or mood instability^12^), which cross diagnostic barriers and can be assessed in subclinical and clinical participants, have also identified associated genetic variants.

Growing evidence from genetic studies suggests that SMI and cardiometabolic diseases (including obesity,^13^ type 2 diabetes,^14^ and coronary artery disease^15^) share pathological mechanisms. Some genetic overlap in the regulation of MDD and platelet phenotypes has been demonstrated, specifically in platelet count, mean platelet volume, and platelet distribution width.^16^ The aim of this study was to build on this work, specifically to determine whether genetic regulation of psychiatric disorders and VTE were correlated and whether genetic predisposition to psychiatric disorders influenced the risk of VTE in the large population-based UK Biobank. Replication analyses were conducted in case-control studies of VTE.

## Methods

### Genetic correlation analyses

Linkage disequilibrium (LD) score regression (using LDSC)^17^ was used to assess the genome-wide genetic overlap between VTE^18^ and MDD,^10^ BD,^9^ or SCZ.^8^ Summary statistics for GWAS meta-analyses of psychiatric illnesses were downloaded from the Psychiatric Genetics Consortium website (https://www.med.unc.edu/pgc/) and those for GWAS meta-analyses of VTE were provided by the INVENT consortium.^19^ Summary statistics from GWAS of research domain criteria traits relevant to MDD, BD, and SCZ were also considered, namely anhedonia,^11^ neuroticism,^20^ and mood instability.^21^ Mood instability is part of the neuroticism score.

### Discovery study population and phenotypes

The UK Biobank study (UKB) has been described in detail.^22^^,^^23^ All participants gave their written informed consent, and this study was conducted with the UKB’s generic approval from the NHS National Research Ethics Service (approval letter dated 29 June 2021, Ref 21/NW/0157).

Briefly, ∼500 000 volunteers were assessed at 22 centers across the United Kingdom between 2006 and 2010. At baseline, individuals underwent physical assessment and blood sampling and completed extensive questionnaires on personal and family medical history, lifestyle, and diet. VTE was defined as self-reported deep vein thrombosis (DVT) and/or pulmonary embolism (PE) (data field #6152). Controls were defined as those answering “none of the above.” Ever smoking (#20116) was defined as current and former smokers vs never smokers. The use of oral contraceptives and hormone replacement therapy was self-reported (#6153). Individuals with missing data or reporting “don’t know” or “prefer not to answer” to any of these questions were excluded. Father, mother, and sibling illness, including MDD, was also recorded (#20107, 20110, and 20111, respectively). A subset of individuals (N ∼150 000) also completed a detailed follow-up questionnaire on mental health, enabling assessment of the lifetime history of MDD, BD, and psychiatric medication use.^24^ A broader category including individuals with a lifetime history of mental illness was constructed.^24^ A family history of MDD was defined as first-degree relatives (parents and siblings) with MDD. Data on a family history of BD or SCZ were not available.

### Genotyping, imputation, and genetic quality control

DNA was extracted from stored blood samples and genotyping, imputation, and standard quality control procedures were conducted centrally by the UKB, as previously described.^22^^,^^23^ Unrelated individuals of self-reported White British ancestry (confirmed using genetic data centrally by the UKB team) were included in this study. In addition, participants were excluded if they were missing more than 10% of their genetic data, had purported sex chromosome aneuploidy, were heterozygosity outliers (as defined by the UKB), or their self-reported sex did not match their genetically determined sex. Genetic principal components (PGCs) were centrally calculated by the UKB team.

### Genotyped construction of the ABO blood groups

ABO blood groups (A, AB, B, and O) were defined as per Garvert et al,^25^ using the genetic variants rs505922, rs8176746, and rs8176747.

### Polygenic risk scores

Polygenic risk scores (PRS)_SCZ_, PRS_BD_, and PRS_MDD_ were based on GWAS meta-analysis results from the Psychiatric Genetics Consortium for SCZ,^8^ BD,^9^ and MDD,^10^ respectively (although the summary statistics used excluded UKB data). PRS was calculated by summing the number of risk-increasing alleles for each individual. There is no consensus on whether it is better to calculate a PRS with fewer strongly associated genetic variants (specific but often low statistical power), all variants across the genome (greater statistical power but a mixture of strongly associated and nonassociated variants), or an intermediate approach. We used LDpred^26^ to calculate the PRS weighted by their effect sizes, including all variants across the genome, as this approach provides more comparable power across the PRS for SCZ, BD, and MDD (108, 30, and 44 significant loci, respectively). LDpred^26^ considers LD patterns based on a reference panel of 1000 UKB participants that were not used in these analyses but passed the genetic quality control above. These weighted PRS were scaled such that the mean was 0 and the standard deviation was 1.

### Discovery statistical analyses in UKB

Logistic regression was used to assess the association of PRS_MDD_, PRS_BD_, and PRS_SCZ_ with VTE. The primary analysis (model 1) was consistent with the models used for previous genetic studies of VTE;^18^ specifically, age, sex, population structure (PGCs 1-8) and technical covariates (genotyping chip) were adjusted. Multiple testing corrections were made for the 3 PRS being analyzed; therefore, *P* value of <.0167 was considered significant. Statistical analyses were conducted using STATA 16.1 (StataCorp, College Station, TX).

Secondary analyses were used to assess the stability of the associations when additional covariates were included: Model 2 (Model 1 plus ABO blood group); Model 3 (Model 2 plus body mass index [BMI], ever smoking, and any antipsychotic medication). PE and DVT were analyzed separately. Additional secondary analyses were conducted, replacing PRS_MDD_ with family history, whereby cases were those with at least one first-degree relative (parents or siblings) with MDD, and controls were those where no first-degree relatives had MDD.

Sensitivity analyses were conducted and first, individuals with any self-reported lifetime experience of mental illness were excluded.^24^ Second, sex-specific analyses were conducted using the models described above and model 4 in women only, where covariates were as for model 3 plus the use of exogenous hormones (hormone replacement therapy and oral contraceptives).

### Replication cohorts

#### RETROVE

RETROVE is a prospective case–control study that included 400 consecutive patients with VTE (cancer-associated thrombosis was excluded) and 400 healthy control volunteers.^27^ All individuals were ≥18 years. The diagnosis was confirmed using Doppler ultrasonography, tomography, magnetic resonance imaging, arteriography, phlebography, or pulmonary gammagraphy. Blood samples from patients were taken ≥6 months after thrombosis to minimize the influence of the acute phase. None of the participants had used oral anticoagulants, heparin, or antiplatelet therapies at the time of blood collection. Controls were selected based on the age and sex distributions of the Spanish population (2001 census). All individuals were genotyped using the Infinium Global Screening Array-24 v3.0 kit from Illumina and imputed using the Haplotype Reference Consortium panel. Written informed consent was obtained from all participants and all procedures were approved by the Institutional Review Board of the Hospital de la Santa Creu i Sant Pau (Barcelona).

GWAS meta-analysis summary statistics from the Psychiatric Genetics Consortium for SCZ,^8^ BD,^9^ and MDD^10^ were used as base data to generate PRS for replication analysis of the RETROVE data (target data). Before downstream analyses, both base and target data were quality-controlled for file transfer, genome build, standard GWAS quality control metrics, mismatching single-nucleotide polymorphisms (SNPs), ambiguous SNPs, duplicated SNPs, sex checks, sample overlap, and relatedness. We computed the PRS from the base and target data using LDPred. The impact of PRS on VTE was tested using logistic regression adjusted for age, sex, and PGCs from 1 to 20. Statistical analyses were performed using R (version 3.5).

#### EOVT

The Early Onset Venous Thrombosis (EOVT) study was a case-control sample of 339 patients with VTE and 1327 healthy French persons from the Suvimax study.^28^ Patients were selected for having documented idiopathic isolated PE or DVT at age <50 years in the absence of acquired risk factors (including surgery, hospitalization, pregnancy, puerperium, oral contraception, cancer, and autoimmune disease) and at the time of VTE and thrombophilia defects (including antithrombin, protein C, or protein S deficiencies, and homozygosity for FV Leiden or FII-20210A). The controls were healthy individuals of European ancestry with no chronic conditions or regular medicine. Genome-wide genotyping and quality control procedures have been previously described.^19^^,^^28^^,^^29^

#### MARTHA

The MARseille THrombosis Association Study (MARTHA) included a sample of 1542 patients with VTE and 1110 healthy individuals.^28^ Patients with VTE were unrelated individuals recruited at the thrombophilia center of La Timone hospital (Marseille) with a history of a first VTE documented by venography, Doppler ultrasound, angiography, and/or ventilation/perfusion lung scans. The patients were free of thrombophilia defects (as in EOVT) and chronic conditions. Controls consisted of 1110 health individuals from the 3C study. Detailed descriptions of these samples and their typing of genome-wide polymorphisms have been extensively described.^19^^,^^29^

#### FARIVE

The Facteurs de Risques et de Récidives de la Maladie Thromboembolique Veineuse (FARIVE) study is a multicenter case–control study composed of 607 patients with a documented episode DVT and/or PE and 607 healthy individuals.^28^ Patients were included if they fulfilled the criteria for a first VTE event, were ≥18 years of age, and had no active cancer or recent history (within 5 years) of malignancy. Controls were matched to cases for age, sex, and center and did not have any history of arterial/venous thrombosis or cancer, liver, or kidney failure. A description of the study can be found in,^28^ and the genotyping procedures and quality controls are available in.^30^

For EOVT, FARIVE, and MARTHA, PRS calculations were performed using PRSice-2 (P + T method), with *P* thresholds of 5 × 10 to 5, 0.05, and 1.0, LD cutoff of r2 + 0.1 and a window of 500 Mb.

#### HVH

The Heart and Vascular Health (HVH) study is a population–based case-control study of risk factors for cardiovascular outcomes set at Group Health (GH), now Kaiser Permanente Washington, in western Washington State. The methods for HVH have been described previously.^18^^,^^31^^,^^32^ For this analysis, VTE cases and controls were utilized; women aged 18 to 89 years were eligible as a VTE case if they experienced a DVT and/or PE between 1995 and 2010, and men aged 30 to 89 years were eligible as a case if they experienced a DVT and/or PE between 2002 and 2010. VTE events were verified by trained medical record abstractors from a review of the complete GH medical record, and controls with no prior history of VTE were selected to meet the same age and identification year as the VTE cases. All study participants were GH members and provided a venous blood sample for DNA extraction. Study approval was granted by the human subjects committee at GH, and informed consent was provided by all study participants.

Two batches of genotyping were completed at the General Clinical Research Center’s Phenotyping/Genotyping Laboratory at Cedars-Sinai: HVH1, using the Illumina 370CNV BeadChip system, and HVH3, using Illumina Omni Express. PRS were created using LDPred2-auto^33^ using the recommended HM3+ SNPs and LD for HVH.

### Replication PRS and analyses

Analyses were consistent with discovery analyses model 1, specifically age, sex, population structure, and any technical variables as covariates. Because of the small size of the replication cohorts, sex-specific analyses were not conducted.

### Meta analyses

Inverse variance-weighted meta-analyses were conducted using STATA 17 (STATA Corp), with Cochran’s Q and I2 used to assess between-study heterogeneity. Meta-analyses were conducted on VTE, as well as DVT and PE separately.

## Results

### Genome-wide genetic correlations between psychiatric traits and VTE

Genome-wide genetic correlation analyses identified a significant overlap for VTE and MDD (Table 1) but not for VTE with BD or SCZ. We further explored the shared genetic correlations between VTE and the endophenotypes of MDD, BD, and SCZ (Table 1), which demonstrated significant correlations with anhedonia and mood instability. These findings consistently suggest that increased genetic liability to increased anhedonia, mood instability, and the risk of MDD is accompanied by an increased risk of VTE.

**Table 1.** **Genetic correlation between VTE and psychiatric illnesses and related endophenotypes**

Source	Psychiatric traits	rg	se	*P* value	*P*_corr value
PGC meta-analysis	MDD	0.179	0.044	**4.36E-05**	**1.31E-04**
PGC meta-analysis	BD	0.056	0.032	7.70E-02	1.39E-01
PGC meta-analysis	SCZ	–0.031	0.038	4.13E-01	4.13E-01
UKB	Anhedonia	0.234	0.043	**4.04E-08**	**3.64E-07**
UKB	Mood instability	0.186	0.037	**3.72E-07**	**1.67E-06**
UKB	Neuroticism	0.071	0.046	1.24E-01	1.39E-01

rg, regression coefficient; se, standard error; *P*_corr, FDR-corrected *P* value.

Boldface indicates significant *P* values.

### Discovery data set: UKB

The characteristics of the UKB are presented in Table 2. In men, 4440 VTE cases were reported, of which the most were DVT (78.4%), and 13.4% reported both DVT and PE. In women there were 6346 VTE cases reported, with 79.1% being DVT and 9.1% reported both DVT and PE. At recruitment, cases were older (59.2 vs 56.8 years) and more overweight (29.3 vs 27.3 kg/m^2^) than the controls. Notably, the age of diagnosis was at least 10 years before recruitment into the study (43.1 years and 45.8 years for PE and DVT respectively). Previous VTE is a contraindication for oral contraceptives and hormone replacement therapy, which (as well as increased age) likely contributed to the reduced prevalence in the case group at recruitment. The frequency of smoking was higher in cases than in controls (50.3% vs 45.6%).

**Table 2.** **Characteristics of the UK Biobank study**

	Men		Women		Combined	
	Cases	Controls	Cases	Controls	Cases	Controls
N (% male)	4440 (100)	135 222 (100)	6346 (0)	149 902 (0)	10 786 (41.2)	285 124 (47.4)
Age (y)	59.5 (7.4)	57.0 (8.1)	59.1 (7.3)	56.6 (7.9)	59.2 (7.4)	56.8 (8.0)
BMI (Kg/m^2^)	29.5 (5.1)	27.8 (4.2)	27.3 (5.4)	26.9 (5.0)	29.3 (5.7)	27.3 (4.7)
Ever Smoker	2608 (59.0)	69 459 (51.6)	2790 (44.2)	60 051 (40.2)	5398 (50.3)	129 510 (45.6)
Oral contraceptives			58 (0.9)	3586 (2.4)	58 (0.9)	3586 (2.4)
HRT			394 (6.3)	10 224 (6.9)	394 (6.3)	10 224 (6.9)
PE	1558 (35.1)		1910 (30.1)		3468 (32.2)	
Age at PE diagnosis	48.8 (12.1)		43.4 (13.2)		45.8 (13.0)	
DVT	3479 (78.4)		5017 (79.1)		8496 (78.8)	
Age at DVT diagnosis	48.4 (11.9)		39.3 (13.7)		43.0 (13.8)	
DVT and PE	597 (13.4)		581 (9.1)		1178 (10.9)	
MDD	177 (4.2)	6184 (4.8)	531 (8.8)	12 977 (9.1)	708 (6.9)	19 161 (7.1)
BD	18 (0.4)	602 (0.5)	32 (0.5)	617 (0.4)	50 (0.5)	1219 (0.4)
GAD	56 (7.7)	1908 (6.4)	189 (17.6)	3615 (11.4)	245 (13.6)	5523 (9.0)

Continuous variables are presented as mean (standard deviation) and categorical variables are presented as number (percentage).

GAD, generalized anxiety disorder; HRT, hormone replacement therapy.

### Primary analyses: impact of psychiatric PRS on VTE risk

As most genetic analyses of VTE were conducted in sex-combined analyses (for increased statistical power), our primary analyses were conducted on sex-combined data (Table 3). PRS_MDD_ was significantly and positively associated with the risk of VTE (model 1: odds ratio [OR], [95% confidence interval (CI)] for 1 SD increase 1.08 [1.06-1.10]; *P* < .001), and secondary analyses demonstrated that this was irrespective of the covariates included. Despite the nonsignificant genetic correlations, for completeness, we also tested PRS_BD_ and PRS_SCZ_ for their association with VTE. PRS_BD_ was also significantly and positively associated with VTE in the sex-combined analyses (model 1: OR [95% CI], 1.03 [1.01-1.06]; *P* = .002), again with secondary analyses demonstrating that this was irrespective of which covariates were included. PRS_SCZ_ demonstrated no effect on the risk of VTE (model 1: [OR (95% CI), 1.00 (0.97-1.02)]; *P* = .688). As mood instability, anhedonia, and neuroticism GWAS were conducted in the UKB/included UKB, we were unable to explore the impact of these traits using PRS, as we have for MDD, BD, and SCZ.

**Table 3.** **Associations between PRS of SCZ, BD, and MDD and the risk of VTE in UKB**

PRS	Model	Sex-combined					Men					Women				
		R2∗	OR	CI	*P* value	N	R2∗	OR	CI	*P* value	N	R2∗	OR	CI	*P* value	N
SCZ	1	0.013	1.00	0.97-1.02	.688	241 483	0.011	0.96	0.93-1.00	.028	114 854	0.012	1.02	0.99-1.05	.182	126 629
	2	0.017	1.00	0.98-1.02	.818	241 470	0.017	0.97	0.93-1.00	.049	114 850	0.015	1.02	0.99-1.05	.164	126 620
	3	0.034	1.00	0.98-1.03	.787	232 482	0.033	0.97	0.93-1.00	.072	111 361	0.033	1.03	0.99-1.06	.057	121 121
	4											0.033	1.03	1.00-1.06	.058	120 685
BD	1	0.013	1.03	1.01-1.06	**.002**	241 483	0.010	1.03	1.00-1.07	.069	114 854	0.012	1.04	1.01-1.07	**.013**	126 629
	2	0.017	1.04	1.01-1.06	**.002**	241 470	0.017	1.03	1.00-1.07	.058	114 850	0.015	1.04	1.01-1.07	**.012**	126 620
	3	0.034	1.04	1.01-1.06	**.002**	232 482	0.030	1.03	1.00-1.07	.073	111 361	0.033	1.04	1.01-1.07	**.011**	121 121
	4											0.033	1.04	1.01-1.07	**.012**	120 685
MDD	1	0.014	1.08	1.06-1.10	**<.001**	284 866	0.012	1.07	1.03-1.10	**<.001**	135 152	0.014	1.09	1.06-1.12	**<.001**	149 714
	2	0.019	1.08	1.06-1.10	**<.001**	284 788	0.018	1.07	1.03-1.10	**<.001**	135 123	0.017	1.09	1.06-1.12	**<.001**	149 665
	3	0.036	1.07	1.04-1.09	**<.001**	274 064	0.034	1.05	1.02-1.09	**.004**	130 864	0.035	1.07	1.04-1.11	**<.001**	143 200
	4											0.035	1.07	1.04-1.11	**<.001**	142 542

Model 1: age, principal genetic components 1 to 8 (PGC1-8) and genotyping chip; model 2: model 1 plus blood group; model 3: model 2 plus BMI, ever smoking and any antipsychotic medication; model 4 (women only), model 3 plus exogenous hormones (hormone replacement therapy and oral contraceptives).

R2∗, pseudo R2.

Boldface indicates singificant *P* values.

### Sensitivity analyses: sex-specific results

Because there was a sex difference in the risk of VTE,^34^ sex-stratified analyses were conducted as sensitivity analyses (Table 3). Although there are sex differences in MDD, it is currently not possible to construct sex-specific PRS_MDD_ due to a lack of sex-specific MDD GWAS meta-analyses. PRS_MDD_ was significantly and positively associated with the risk of VTE in both men and women (model 1: OR [95% CI], 1.07 [1.03-1.10]; *P* < .001 and 1.09 [1.06-1.12]; *P* < .001, respectively). The secondary analyses demonstrated that this was irrespective of the covariates included. Inclusion of sex in the PRS_MDD_ interaction term demonstrated a nonsignificant result (Models 1-3; *P* = .049 to .190). PRS_BD_ demonstrated a nominal positive association with VTE in women, irrespective of the covariates used (model 1: OR [95% CI], 1.04 [1.01-1.07]; *P* = .013). No association was observed between PRS_BD_ and VTE in the men. However, there was no evidence of a sex-PRS_BD_ interaction (models 1-3; *P* = .573 to .599). PRS_SCZ_ showed no significant association with VTE in men or women; however, the opposite effects were noted. This was confirmed with a sex by PRS_SCZ_ interaction being observed (Models 1-3; *P* = .004 to .005).

### Secondary analyses: exclusion of individuals with self-reported mental illness

To assess whether the associations between PRS_BD_ and PRS_MDD_ were driven by individuals with a lifetime experience of psychiatric conditions, analyses were also conducted, excluding all individuals who self-reported any mental illness (broad definition^24^). Demographics of those without mental illness were consistent with those observed in the entire cohort (supplemental Table 1), with VTE cases being older (59.4 vs 56.8 years), having a higher BMI (29.1 vs 27.2 kg/m^2^) and being more likely to smoke (49.3% vs 45.0%) than the controls. The sex-combined results were similar to those in the main analyses (supplemental Table 2), with PRS_BD_ and PRS_MDD_ being associated with an increased risk of VTE (model 1: OR [95% CI], 1.04 [1.01-1.06]; *P* = .004 and 1.06 [1.04-1.09]; *P* < .001, respectively).

### Sensitivity analyses: family history instead of PRS_MDD_

As genetic data are currently not clinically available, we compared whether family history confers similar information to PRS_MDD_. Therefore, further sensitivity analysis replaced PRS_MDD_ with a family history (first-degree relatives) of MDD. Similar results were observed in which a family history of MDD was associated with an increased risk of VTE (supplemental Table 3). Stronger effects observed using family history are expected, as family history includes environmental and behavioral patterns, as well as genetics, whereas PRS_MDD_ only reflects genetic variation effects. Family history of BD or SCZ were not available.

### Replication of psychiatric PRS on VTE risk: replication and sex-combined

**Figure 1.**
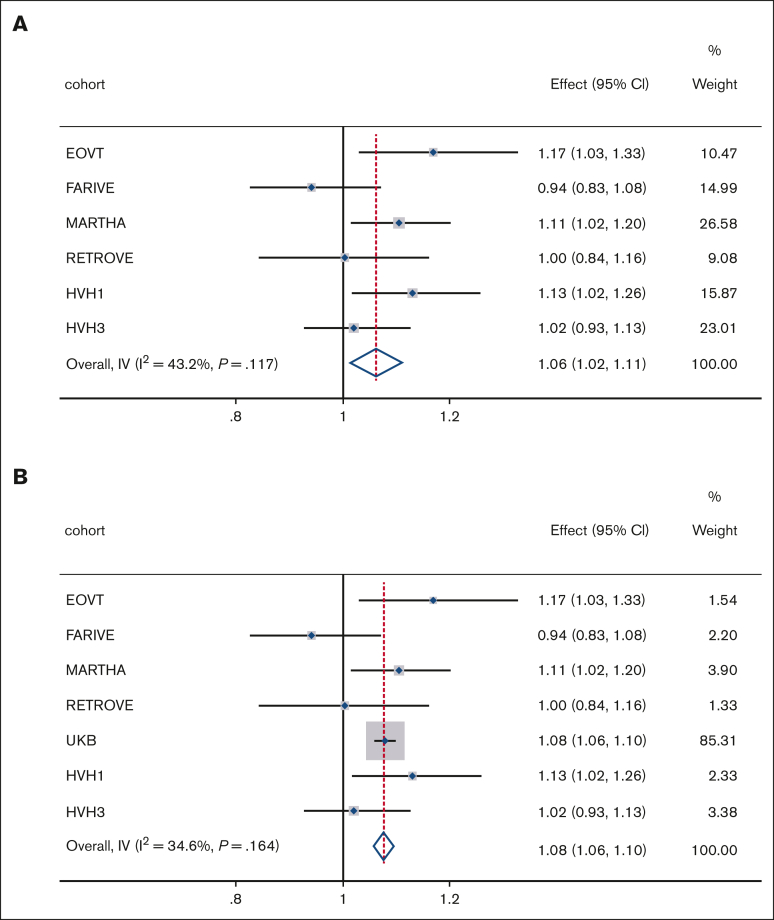
**Effect of PRS_MDD_ on VTE risk.** Meta-analyses results of (A) PRS_MDD_ in replication cohorts and (B) PRS_MDD_ in discovery and replication cohorts. Effect, odds ratio; weight, relative weight in the analyses based on sample size; *I*^2^, measure of heterogeneity; IV, inverse variance; *P*, *P* value for heterogeneity measure.

The descriptive statistics of the replication studies are presented in supplemental Table 4. Analysis of the replication studies was restricted to sex-combined analyses (model 1). Individual cohort results are presented in supplemental Table 5. Meta-analyses of replication cohorts demonstrated a significant positive effect of PRS_MDD_ on VTE risk (Figure 1A), with consistent results being observed when the discovery and replication results were combined (Figure 1B). In contrast, no association was observed for PRS_BD_, which demonstrated very high heterogeneity between studies (supplemental Figures 1A-B). No association was observed for PRS_SCZ_. To explore heterogeneity, we conducted meta-analyses of DVT and PE separately (this was not possible in RETROVE). PRS_MDD_ was positively associated with DVT in all cohorts (supplemental Figure 2A), although there was some variability in the association between PRS_MDD_ and PE. Mixed null results were observed for PRS_BD_ vs PRS_SCZ_ and DVT or PE (supplemental Figure 2C-F).

## Discussion

This study demonstrated that the genetic regulation of VTE overlaps with that of MDD and that increased genetic predisposition (as measured using PRS) to MDD was associated with an increased risk of VTE, which was independent of major clinical risk factors and not driven by individuals with lifetime experience of self-reported mental illness. No associations were observed for BD or SCZ.

In the clinical context, the risks of VTE (also in UKB) associated with monogenic thrombophilia variants, as well as PRS based on the largest GWAS of VTE (PRS_VTE_) to date, have been described.^35^ Compared with our PRS_MDD_ (VTE OR = 1.08), the risk of VTE associated with carriers of 1 or 2 F5 R534Q alleles (OR = 2.21 and 7.3, respectively), 1 or 2 F2 G20210A alleles (OR = 1.74 and 3.31, respectively), or 1 allele of each (OR = 3.94) was much higher.^35^ Similarly, the top decile of PRS_VTE_ had a greater impact on VTE risk (OR = 2.35).^35^

MDD, BD, and SCZ share some symptoms and genetic regulation; therefore, it was interesting that the genetic correlation observed between VTE and MDD was not observed for BD and SCZ. This could be because of the sample size and power of the underlying GWAS (MDD is more prevalent than SCZ or BD). Because anhedonia is a key feature of MDD, it was reassuring that the VTE-anhedonia genetic correlation was consistent with that of MDD. The greater strength of the correlation might reflect a less heterogeneous phenotype used in the GWAS of anhedonia compared with the notoriously heterogeneous MDD diagnosis. It should be noted that the moderate regression coefficients are likely to be underestimated because the VTE analyses are transancestry analysis (European and African ancestry), whereas the MDD, anhedonia, and mood instability analyses are of European ancestry only. Although rare variants associated with monogenic thrombophilias are more common in the African ancestry than in European ancestry, they are likely excluded from the European GWAS (due to very low minor allele frequency). Furthermore, differences in LD structure, allele frequencies, and effect sizes between the European MDD and European/African VTE analyses would bias the results toward the null (smaller regression coefficients) rather than causing inflation/false positive effects.

The stronger association with DVT than with PE likely reflects the sample size of these diagnoses (especially in the replication cohorts), as the genetic correlation between DVT and PE has been shown to be very high^35^ and a systematic review of VTE in psychiatric disorders^4^ provided no evidence for a differential effect of phenotypic MDD on the risk of DVT vs PE. Thus, any differences in the associations between PRS_MDD_, DVT, and PE would probably reflect the differential effects of environmental risk factors or the interaction between genetic and environmental risk factors.

Sex-specific analyses demonstrated a significant association between PRS_BD_ and VTE in men but not in women; however, it was not possible to replicate the smaller size of the replication data sets precluding the use of stratified analyses. In addition, GWAS of BD is the smallest of the psychiatric traits, which limits the ability to detect genetic associations with BD. When larger GWAS of BD are available, it would be worth reassessing the association between PRS_BD_ and VTE, especially considering sex-specific effects.

The stability of the association between PRS_MDD_ and VTE in UKB, irrespective of covariates used, suggests that PRS_MDD_ represents an independent mechanism, not captured by existing risk factors. Future studies should consider whether the association between PRS_MDD_ and VTE occurs via risk factors such as immobilization, hospitalization, surgery, and cancer; the UKB study does have data on these variables, but as their timing in relation to the VTE event is unclear, attempting to consider them here could provide misleading results. Further studies are required to identify the mechanism(s) through which PRS_MDD_ promotes VTE. Whether the relationship between PRS_MDD_ and VTE is causal remains an obvious question; however, the assumptions underlying Mendelian randomization methods mean that the use of genetic variation associated with a binary trait is not recommended.^36^ Future studies should investigate continuous measures of relevance to MDD to address this question.

A family history of MDD is associated with an increased risk of VTE and has important implications for translation into clinical practice. Genetic data are not available for clinical purposes; however, asking a patient about MDD in their first-degree relatives is feasible in a clinical setting. Family history encompasses environmental and genetic risk factors, meaning that the larger effects observed for family history are unsurprising. Additional research is required to assess whether the inclusion of a family history of MDD in VTE risk prediction models would be beneficial. The same analyses were not possible for BD and SCZ, as their family history was not collected.

A strength of this study is that the results are consistent between the population-based UKB study that used self-reports of VTE and 6 clinical studies with more rigorous case–control definitions, with the meta-analysis of the clinical studies (Figure 1A) demonstrating that the association is not driven by UKB alone. Furthermore, although UKB has a well-recognized healthy participant bias,^37^ the 6 replication cohorts were collected from 3 different countries worldwide, with differing clinical inclusion and exclusion criteria, and differing burdens of risk factors such as non-O blood group, BMI, smoking, antipsychotic medication, and exogenous hormones. This consistency suggests that the observed association was robust. The limitations of this study include the fact that the discovery of case-control status relied upon self-reporting. Although not perfect, previous studies have demonstrated that self-reporting of VTE is reliable and provides findings similar to those that are more rigorously defined.^38^ In addition, the retrospective nature of the discovery data is challenging, as covariates such as smoking and BMI were recorded at recruitment, not at the time of diagnosis; therefore, this study assumed that these variables do not change over time, which may not be valid. Finally, PRS does not generalize well to additional ancestry groups (with that of the GWAS upon which they are based), which hinders our assessment of this association in individuals of non-White British ancestry in the UKB.

In summary, we present evidence that a genetic predisposition to MDD is an independent risk factor for VTE. Further studies are required to understand the mechanisms underlying the association between predisposition to MDD and VTE. Similar results were observed when considering family history (first-degree relatives) of MDD instead of PRS_MDD_; therefore, in the absence of GWAS data for individuals, considering family history of mental illness could be considered when assessing the risk of VTE.

Conflict-of-interest disclosure: The authors declare no competing financial interests.
