# Supplementary material for: Polygenic risk of major depressive disorder as a risk factor for venous thromboembolism
Source: Blood Adv. 2023 Jul 6;7(18):5341–50. doi: 10.1182/bloodadvances.2023010562 (PMC10506044; doi:10.1182/bloodadvances.2023010562)
Supplement: Supplemental Tables and Figures [file BLOODA_ADV-2023-010562-mmc1.docx]

**Supplemental Data**

Supplemental Figure 1: Meta-analyses results of A) PRS_BD_ in replication cohorts and B) PRS_BD_ in discovery and replication cohorts, C) PRS_SCZ_ in replication cohorts, D) PRS_SCZ_ in discovery and replication cohorts. Where: Effect, odds ratio; 95%CI, 95% confidence interval; weight, relative weight in the analyses, based on sample size; I^2^, measure of heterogeneity; p, p-value for heterogeneity measure.

Supplemental Figure 2: Meta-analyses results of A) PRS_MDD_ and DVT, B) PRS_MDD_ and PE, C) PRS_BD_ and DVT, D) PRS_BD_ and PE, E) PRS_SCZ_ and DVT and F) PRS_SCZ_ and PE in discovery and replication cohorts. Where: Effect, odds ratio; 95%CI, 95% confidence interval; weight, relative weight in the analyses, based on sample size; I^2^, measure of heterogeneity; p, p-value for heterogeneity measure.

| Supplementary Table 1: Characteristics of the UK Biobank study without mental illness | | | | | |  |
| --- | --- | --- | --- | --- | --- | --- |
|  | Men | | Women | | Combined | |
|  | cases | controls | cases | controls | cases | controls |
| N (% male) | 3866 (100) | 124458 (100) | 5130 (0) | 131701 (0) | 8996 (43.0) | 256159 (48.6) |
| Age (years) | 59.6 (7.4) | 57.0 (8.1) | 59.3 (7.3) | 56.6 (8.0) | 59.4 (7.3) | 56.8 (8.10) |
| BMI (Kg/m2) | 29.4 (5.0) | 27.8 (4.1) | 28.8 (5.8) | 26.7 (4.9) | 29.1 (5.5) | 27.2 (4.6) |
| Ever Smoker | 2243 (58.3) | 63134 (50.9) | 2171 (42.5) | 51576 (39.3) | 4414 (49.3) | 114710 (45.0) |
| Oral contraceptives |  |  | 48 (0.01) | 3233 (2.5) |  |  |
| HRT |  |  | 301 (5.9) | 8476 (6.5) |  |  |
| PE | 1339 (34.6) |  | 1495 (29.1) |  | 2834 |  |
| Age at PE diagnosis | 48.6 (12.3) |  | 43.6 (13.2) |  | 45.9 (13.0) |  |
| DVT | 3040 (78.6) |  | 4065 (79.2) |  | 7105 |  |
| Age at DVT diagnosis | 48.5 (11.9) |  | 39.4 (13.7) |  | 43.3 (13.7) |  |
| DVT and PE | 513 (13.3) |  | 430 (8.4) |  | 943 |  |
| Where: HRT, hormone replacement therapy; PE, pulmonary embolism; DVT, deep vein thrombosis | | | | | | |

| Supplementary Table 2: Associations between PRS and risk of VTE in individuals without mental illness | | | | | | | | | | | | | | |  |  |  |
| --- | --- | --- | --- | --- | --- | --- | --- | --- | --- | --- | --- | --- | --- | --- | --- | --- | --- |
|  |  | men | | | | | women | | | | | sex-combined | | | | |  |
| PRS | Model | R2* | OR | CI | P | N | R2* | OR | CI | P | N | R2* | OR | CI | P | N |  |
| SCZ | 1 | 0.011 | 0.96 | 0.92-0.99 | **0.015** | 105471 | 0.013 | 1.01 | 0.97-1.04 | 0.701 | 110563 | 0.01 | 0.98 | 0.96-1.01 | 0.183 | 216034 |  |
|  | 2 | 0.018 | 0.96 | 0.92-1.00 | 0.260 | 105467 | 0.160 | 1.01 | 0.98-1.04 | 0.659 | 110556 | 0.02 | 0.99 | 0.96-1.01 | 0.251 | 216023 |  |
|  | 3 | 0.033 | 0.97 | 0.93-1.00 | 0.075 | 104747 | 0.030 | 1.02 | 0.99-1.05 | 0.286 | 109899 | 0.03 | 1.00 | 0.97-1.02 | 0.712 | 214646 |  |
|  | 4 |  |  |  |  |  | 0.031 | 1.02 | 0.98-1.05 | 0.298 | 109517 |  |  |  |  |  |  |
| BD | 1 | 0.110 | 1.04 | 1.00-1.07 | 0.550 | 105471 | 0.013 | 1.04 | 1.00-1.07 | 0.031 | 110563 | 0.01 | 1.04 | 1.01-1.06 | **0.004** | 216034 |  |
|  | 2 | 0.018 | 1.04 | 1.00-1.08 | 0.046 | 105467 | 0.160 | 1.04 | 1.00-1.07 | 0.030 | 110556 | 0.02 | 1.04 | 1.01-1.06 | **0.003** | 216023 |  |
|  | 3 | 0.034 | 1.04 | 1.00-1.08 | 0.030 | 104747 | 0.030 | 1.04 | 1.00-107 | 0.240 | 109899 | 0.03 | 1.04 | 1.01-1.06 | **0.002** | 214646 |  |
|  | 4 |  |  |  |  |  | 0.031 | 1.04 | 1.00-1.07 | 0.270 | 109517 |  |  |  |  |  |  |
| MDD | 1 | 0.122 | 1.05 | 1.01-1.09 | **0.016** | 124206 | 0.150 | 1.07 | 1.04-1.11 | **<0.001** | 131157 | 0.01 | 1.06 | 1.04-1.09 | **<0.001** | 255363 |  |
|  | 2 | 0.018 | 1.05 | 1.01-1.09 | **0.018** | 124177 | 0.018 | 1.07 | 1.04-1.01 | **<0.001** | 131112 | 0.02 | 1.06 | 1.03-1.09 | **<0.001** | 255289 |  |
|  | 3 | 0.034 | 1.04 | 1.00-1.08 | 0.051 | 123138 | 0.032 | 1.06 | 1.03-1.10 | **<0.001** | 150213 | 0.03 | 1.05 | 1.03-1.08 | **<0.001** | 253351 |  |
|  | 4 |  |  |  |  |  | 0.032 | 1.06 | 1.03-1.10 | **<0.001** | 129626 |  |  |  |  |  |  |
| VTE | 1 | 0.040 | 1.63 | 1.57-1.70 | **<0.001** | 103603 | 0.027 | 1.38 | 1.34-1.42 | **<0.001** | 108458 | 0.03 | 1.48 | 1.45-1.52 | **<0.001** | 212061 |  |
|  | 2 | 0.042 | 1.76 | 1.69-1.83 | **<0.001** | 103600 | 0.027 | 1.46 | 1.40-1.51 | **<0.001** | 108454 | 0.03 | 1.58 | 1.54-1.63 | **<0.001** | 212054 |  |
|  | 3 | 0.058 | 1.76 | 1.69-1.84 | **<0.001** | 102904 | 0.041 | 1.46 | 1.40-1.51 | **<0.001** | 107818 | 0.05 | 1.58 | 1.54-1.63 | **<0.001** | 204619 |  |
|  | 4 |  |  |  |  |  | 0.04 | 1.46 | 1.41-1.52 | **<0.001** | 107456 |  |  |  |  |  |  |
| Where: R2*, Pseudo R2; Model 1: age, principle genetic components 1-8 (PGC1-8) and genotyping chip; Model 2: Model 1 plus blood group; Model 3: model 2 plus BMI, ever smoking and any anti-psychotic medication; Model 4 (women only), model 3 plus exogenous hormones (hormone replacement therapy and oral contraceptives) | | | | | | | | | | | | | | | | |  |
|  |  |  |  |  |  |  |  |  |  |  |  |  |  |  |  |  |  |
|  |  |  |  |  |  |  |  |  |  |  |  |  |  |  |  |  |  |

| Supplementary Table 3: Effect of family history of MDD on VTE risk. | | | | | | | | | |  |  |  |  |
| --- | --- | --- | --- | --- | --- | --- | --- | --- | --- | --- | --- | --- | --- |
|  | men (N=120724) | | | | | women | | | | sex-combined (N= 257487) | | | |
| Model | R2* | OR | CI | P | R2* | | OR | CI | P | R2* | OR | CI | P |
| 1 | 0.012 | 1.19 | 1.08-1.32 | **<0.0001** | 0.015 | | 1.29 | 1.20-1.39 | **<0.0001** | 0.015 | 1.26 | 1.19-1.33 | **<0.0001** |
| 2 | 0.019 | 1.20 | 1.09-1.33 | **<0.0001** | 0.018 | | 1.29 | 1.20-1.39 | **<0.0001** | 0.020 | 1.26 | 1.19-1.33 | **<0.0001** |
| 3 | 0.036 | 1.20 | 1.09-1.33 | **<0.0001** | 0.034 | | 1.27 | 1.18-1.37 | **<0.0001** | 0.036 | 1.24 | 1.17-1.32 | **<0.0001** |
| 4 |  |  |  |  | 0.035 | | 1.27 | 1.17-1.36 | **<0.0001** |  |  |  |  |
| Where: R2*, Pseudo R2; Model 1: age, principle genetic components 1-8 (PGC1-8) and genotyping chip; Model 2: Model 1 plus blood group; Model 3: model 2 plus BMI, ever smoking and any anti-psychotic medication; Model 4 (women only), model 3 plus exogenous hormones (hormone replacement therapy and oral contraceptives) | | | | | | | | | | | | | |

| STable 4: Descriptive statistics of the replication cohorts | | | | | | | | | | | | |
| --- | --- | --- | --- | --- | --- | --- | --- | --- | --- | --- | --- | --- |
|  | RETROVE | | HVH1 | | HVH3 | | EOVT | | FARIVE | | MARTHA | |
|  | cases | controls | cases | controls | cases | controls | cases | controls | cases | controls | cases | controls |
| Sex (%), male | 196 (49.0) | 194 (48.50) | 716 (4.61) | 877 (30.9) | 976 (54.61) | 757 (40.16) | 229 (56) | 370 (30) | 241 (40) | 259 (43) | 522 (34) | 522 (34) |
| Age (SD) | 61.73 (18.56) | 47.09 (18.18) | 66.24 (12.12) | 66.88 (9.33) | 61.01 (15.24) | 63.55 (13.09) | 35.19 (11.25) |  | 53.16 (19.65) | 51.46 (18.56) | 40.94 (15.70) | 68.07 (2.24) |
| BMI (SD), kg/m2 | 28.08 (5.22) | 25.36 (4.08) | 31.68 (8.17) | 29.43 (6.31) | 31.51 (7.53) | 30.12 (6.49) |  |  | 26.35 (5.85) | 25.87 (6.04) | 26.02 (4.81) |  |
| Ever Smoker (yes) | 55 (13.75) | 75 (18.75) | 299 (41.76) | 440 (50.17) | 499 (51.13) | 395 (52.18) |  |  | 294 (48.43) | 336 (55.44) | 157 (10.18) |  |
| Exogenous hormones | 73 (35.78) | 125 (60.68) | 25  (3.49) | 2  (0.23) | 72  (7.38) | 19  (2.51) |  |  |  |  |  |  |
| HRT | 9 (4.43) | 23 (11.17) | 198 (27.65) | 184 (20.98) | 21  (2.15) | 64  (8.45) |  |  | 47 (12.84) | 50 (14.37) |  |  |
| PE | 181 (45.25) | 0 (0) | 234 (32.7) |  | 294 (30.1) |  | 143 (42) |  | 245(40) |  | 44 (7.7) |  |
| Age at PE diagnosis |  |  | 67.02 (11.56) |  | 62.38 (16.03) |  |  |  |  |  |  |  |
| DVT | 280 (70.00) | 0 (0) | 365 (51.0) |  | 476 (48.8) |  | 196 (58) |  | 173(29) |  | 444 (78) |  |
| Age at DVT diagnosis |  |  | 65.89 (12.49) |  | 59.59 (14.89) |  |  |  |  |  |  |  |
| DVT and PE | 70 (17.50) | 0 (0) | 117 (16.3) |  | 206 (21.1) |  |  |  | 188 (31) |  | 81 (14) |  |
| Blood group (type O) | 93 (23.48) | 153 (38.54) | 234 (32.68) | 374 (42.65) | 301 (30.84) | 342 (45.18) |  |  | 157 (30) | 239 (45) |  |  |
| anti-psychotics | 16  (4.00) | 7  (1.75) | 23  (3.21) | 3  (0.34) | 19  (1.95) | 2  (0.26) |  |  |  |  |  |  |

| STable 5: Individual replication cohort results for VTE | | | | | | | |  |  |  |  |  |  |  |  |  |  |  |  |  |  |  |  |  |  |
| --- | --- | --- | --- | --- | --- | --- | --- | --- | --- | --- | --- | --- | --- | --- | --- | --- | --- | --- | --- | --- | --- | --- | --- | --- | --- |
| PRS | RETROVE | |  |  | HVH1 |  |  |  | HVH3 |  |  |  | P threshold | EOVT |  |  |  | FARIVE | |  |  | MARTHA | |  |  |
|  | OR | L95 | U95 | P | OR | L95 | U95 | P | OR | L95 | U95 | P |  | OR | L95 | U95 | P | OR | L95 | U95 | P | OR | L95 | U95 | P |
| BD |  | | | | | | | | | | | | 5e.05 | 1.15 | 1.01 | 1.30 | **0.0320** | 0.98 | 0.86 | 1.12 | 0.7741 | 1.04 | 0.96 | 1.13 | 0.3361 |
|  |  |  |  |  |  |  |  |  |  |  |  |  | 0.05 | 1.68 | 1.47 | 1.93 | **1.30E-13** | 0.84 | 0.74 | 0.96 | **0.0109** | 1.03 | 0.94 | 1.12 | 0.5298 |
|  | 0.99 | 0.97 | 1.01 | 0.4078 | 0.935 | 0.839 | 1.043 | 0.2280 | 0.947 | 0.858 | 1.046 | 0.283 | 1 | 1.82 | 1.59 | 2.10 | **2.97E-17** | 0.85 | 0.74 | 0.97 | **0.0130** | 1.07 | 0.98 | 1.17 | 0.1484 |
| SCZ |  | | | | | | | | | | | | 5e.05 | 1.02 | 0.87 | 1.19 | 0.8529 | 1.00 | 0.88 | 1.14 | 0.9923 | 1.08 | 0.98 | 1.19 | 0.1126 |
|  |  |  |  |  |  |  |  |  |  |  |  |  | 0.05 | 1.06 | 0.93 | 1.22 | 0.3622 | 1.05 | 0.92 | 1.19 | 0.4982 | 1.09 | 1.00 | 1.19 | 0.0664 |
|  | 1.00 | 0.98 | 1.01 | 0.3311 | 0.893 | 0.803 | 0.993 | 0.0375 | 0.979 | 0.887 | 1.08 | 0.666 | 1 | 1.10 | 0.96 | 1.25 | 0.1750 | 1.00 | 0.88 | 1.15 | 0.9524 | 1.06 | 0.97 | 1.16 | 0.2252 |
| MDD |  | | | | | | | | | | | | 5e.05 | 0.98 | 0.86 | 1.12 | 0.7717 | 0.94 | 0.82 | 1.06 | 0.3105 | 1.01 | 0.93 | 1.10 | 0.8085 |
|  |  |  |  |  |  |  |  |  |  |  |  |  | 0.05 | 1.15 | 1.01 | 1.30 | **0.0327** | 0.93 | 0.82 | 1.06 | 0.2892 | 1.12 | 1.03 | 1.21 | **0.0107** |
|  | 1.12 | 0.79 | 1.44 | 0.5059 | 1.131 | 1.017 | 1.259 | 0.0237 | 1.022 | 0.927 | 1.128 | 0.657 | 1 | 1.17 | 1.03 | 1.33 | **0.0153** | 0.94 | 0.83 | 1.08 | 0.3762 | 1.11 | 1.02 | 1.20 | **0.0197** |
| Where: RETROVE, HVH1 and HVH3 PRS were calculated using LDPred (includes all SNPs). RETROVE adjusted for age, sex, principle components 1-20. HVH1 and HVH3 adjusting for age, sex and PCs 1-2. | | | | | | | | | | | | | | | | | | | | | | | | | |
