# Supplementary figures and images for: Polygenic risk of major depressive disorder as a risk factor for venous thromboembolism
Source: Blood Adv. 2023 Jul 6;7(18):5341–50. doi: 10.1182/bloodadvances.2023010562 (PMC10506044; doi:10.1182/bloodadvances.2023010562)

SFigure 1

A)

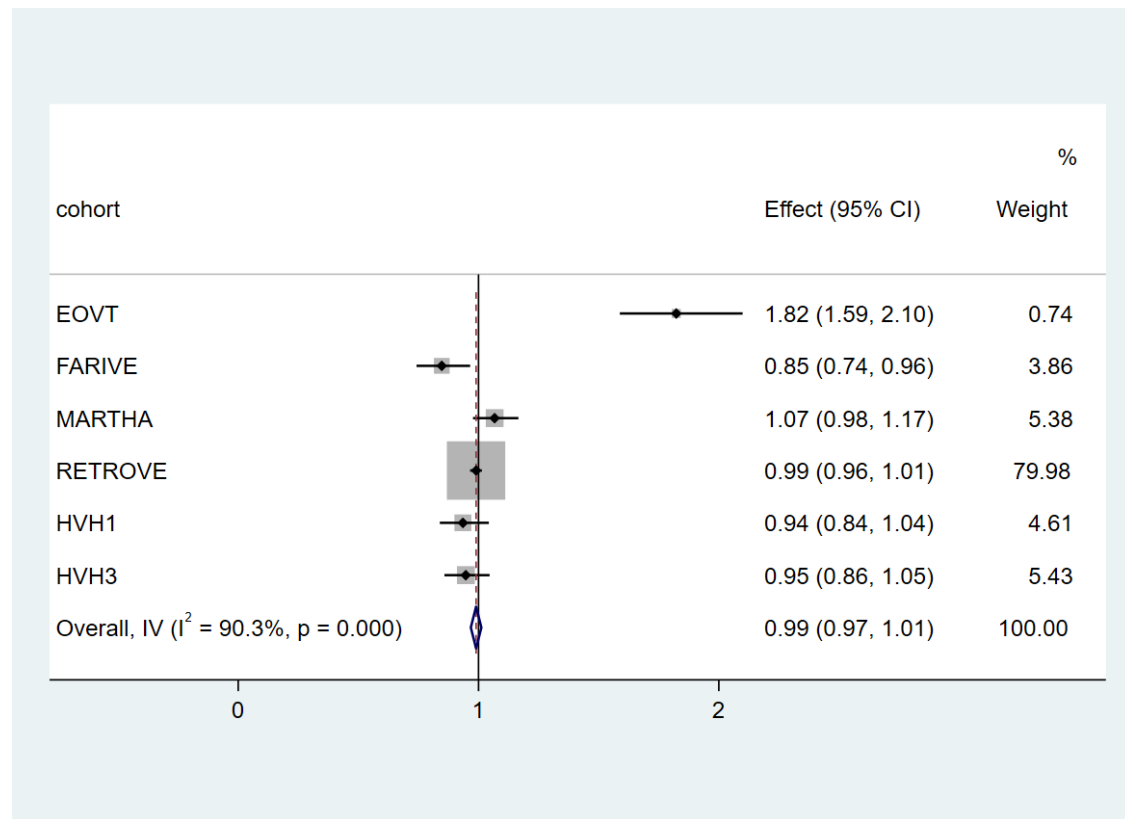

B)

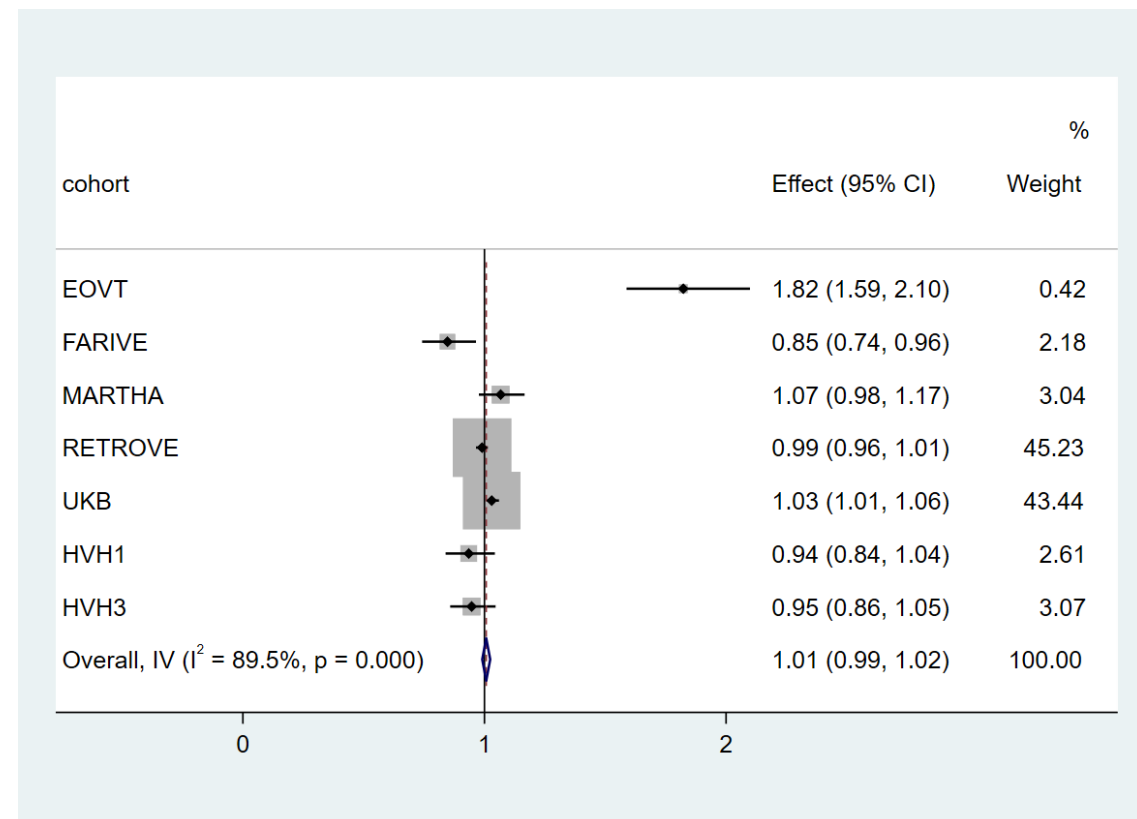

C)

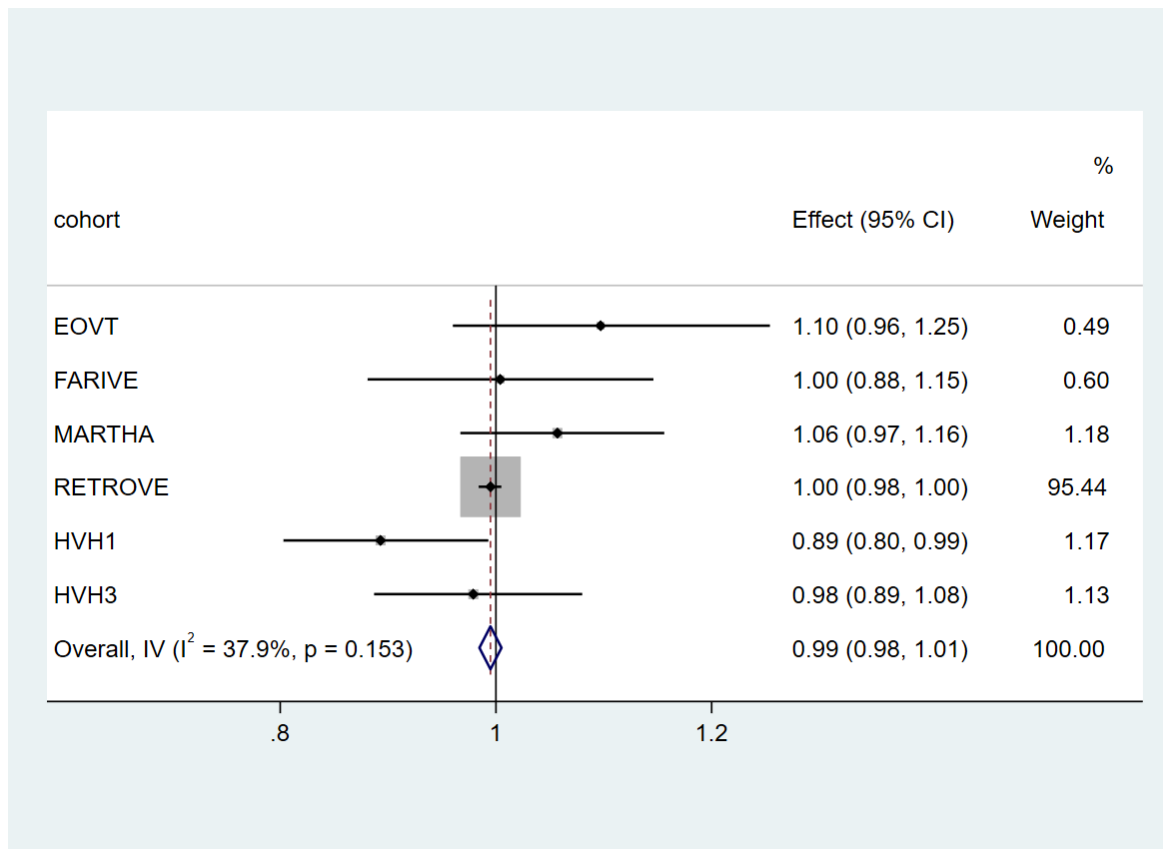

D)

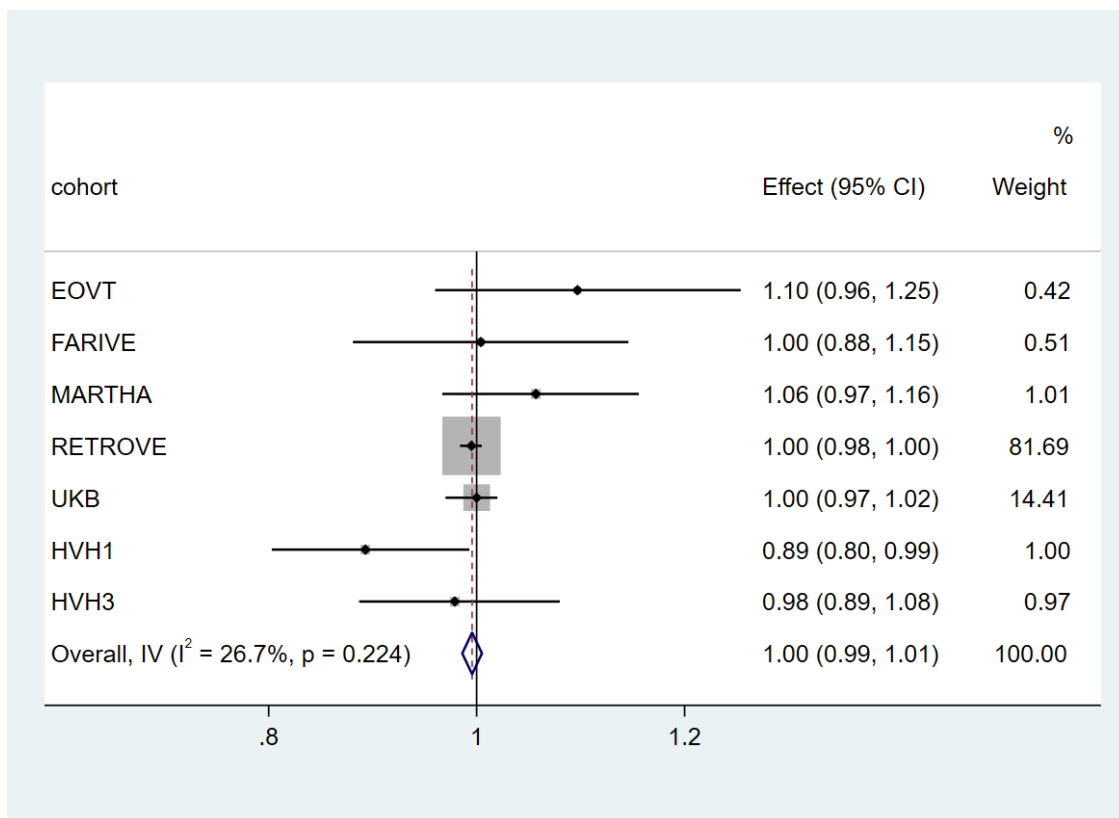

Supplement: Supplemental Figure 1 [file BLOODA_ADV-2023-010562-mmc2.pdf]

Figure 2

A)

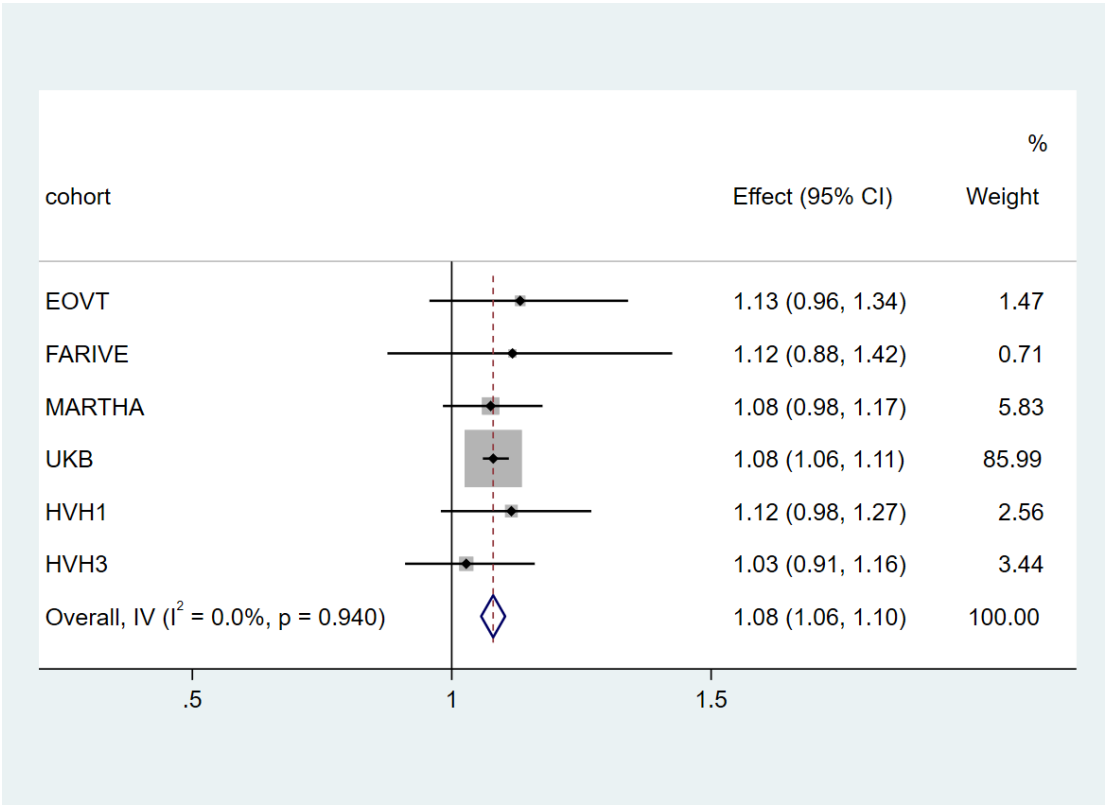

B)

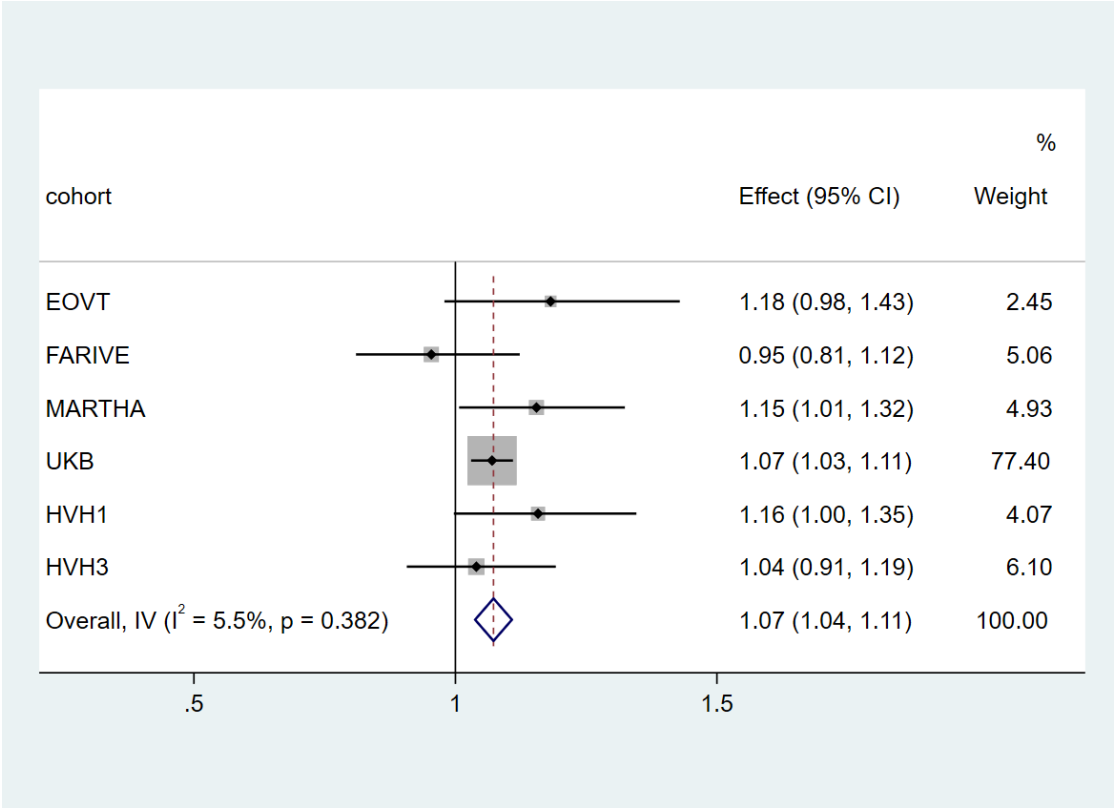

C)

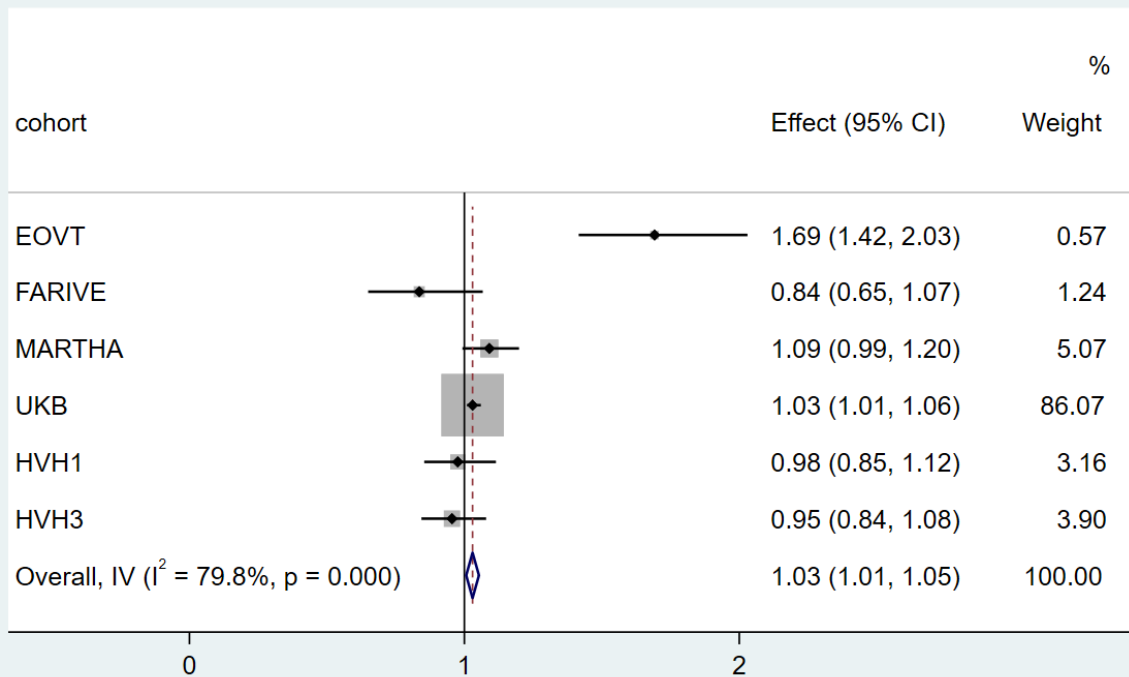

D)

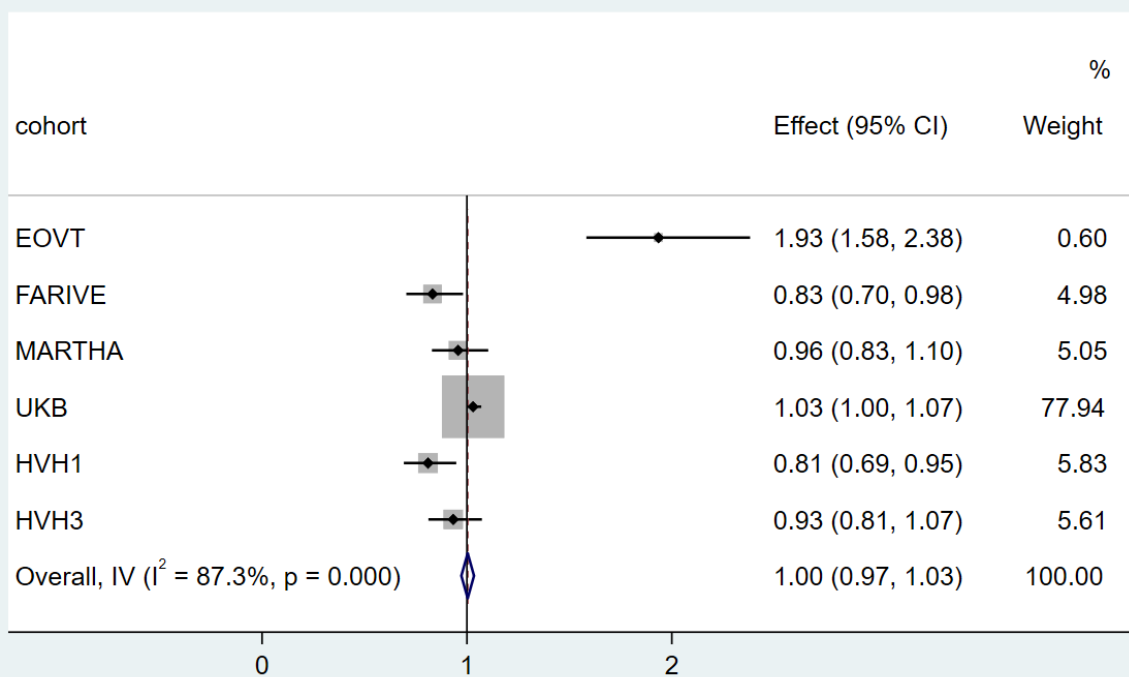

E)

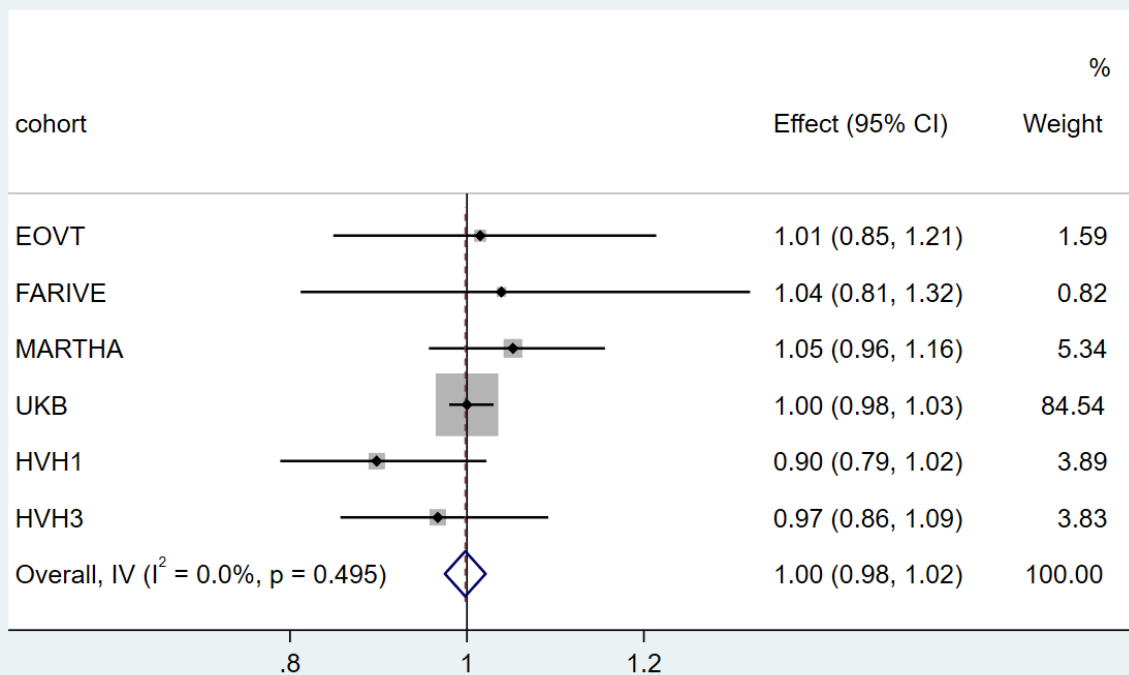

F)

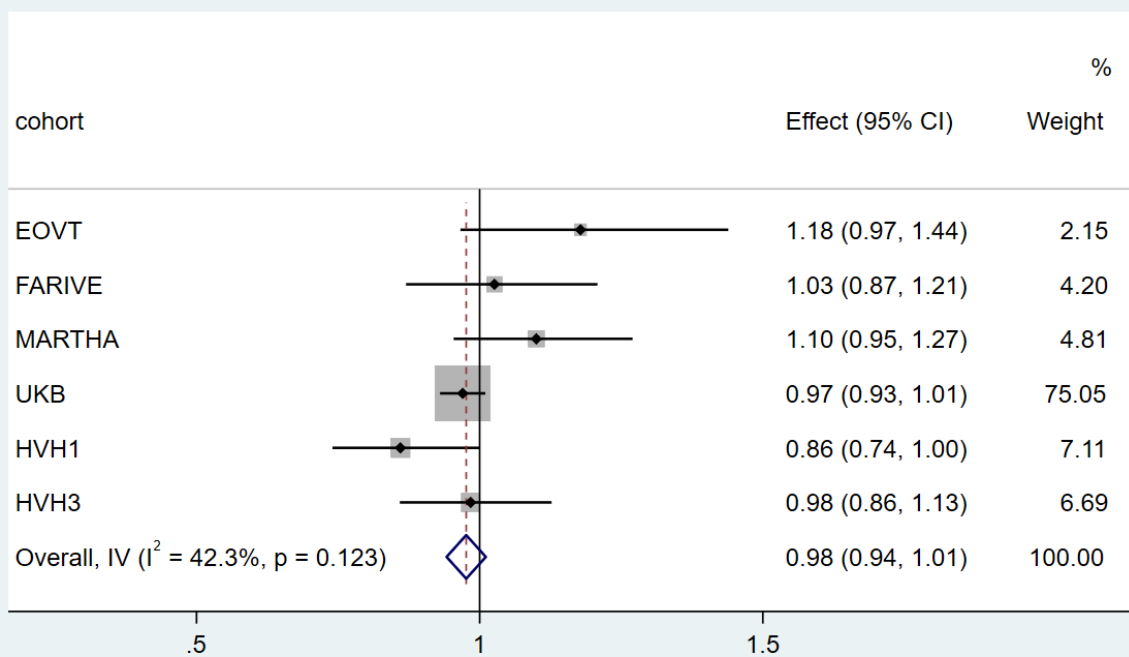

Supplement: Supplemental Figure 2 [file BLOODA_ADV-2023-010562-mmc3.pdf]
